# Cronkhite-Canada syndrome: A case report and literature review

**DOI:** 10.1097/MD.0000000000040242

**Published:** 2024-10-25

**Authors:** Nanping Wang, Yue Xiang, Liping Tao, Wen Ming, Lijun Ren, Tao Huang, GuoDong Yang, Jian Gao, Quan Ren, Peng Zhu, Min Huang

**Affiliations:** a Department of Gastroenterology, Affiliated Hospital of North Sichuan Medical College, Nanchong, Sichuan, China; b The North Sichuan Medical College, Nanchong, Sichuan, China; c Department of Gastroenterology, The Second Affiliated Hospital of ChongQing Medical University, ChongQing, China; d Department of Pathology, Affiliated Hospital of North Sichuan Medical College, Nanchong, Sichuan, China; e Department of Geriatric Medicine, Affiliated Hospital of North Sichuan Medical College, Nanchong, Sichuan, China; f Department of General Surgery, Nanchong Gaoping District People’s Hospital, Nanchong, Sichuan, China.

**Keywords:** Cronkhite-Canada syndrome (CCS), eosinophils, gastrointestinal polyps, mast cells, treatment

## Abstract

**Rationale:**

Cronkhite-Canada syndrome (CCS) is a nonhereditary, rare polyposis condition, first documented by Cronkhite and Canada in 1955. The primary distinct features of this syndrome include ectodermal abnormalities and diffuse gastrointestinal polyp changes accompanied by protein loss. The primary clinical manifestations of CCS include hair loss, excessive pigmentation of the skin, and malnourishment of fingernails or toenails. Other notable symptoms include weight loss, protein-losing enteropathy, diarrhea, abdominal pain, nausea, vomiting, taste abnormalities, and atrophic glossitis, which predominantly occur in middle-aged and older males. CCS is characterized by an extremely rare, nonfamilial hamartomatous polyposis syndrome, in which polyps are distributed in the stomach and colon (90%), small intestine(80%), and rectum (67%), while sparing the esophagus.

**Patient concerns:**

This report describes a 72-year-old female, initially treated for intestinal obstruction, followed by a small intestine resection. She reported diarrhea, emaciation, and loss of appetite across various hospitals.

**Diagnoses:**

Endoscopic examination of the stomach and colon, plus capsule endoscopy, revealed multiple polyps throughout her gastrointestinal tract, except in the esophagus.

**Interventions:**

Treatment included hormones with antiallergic medication, acid-suppressing drugs, salicylates, and nutritional support with zinc sulfate, adding trace elements and amino acids.

**Outcomes:**

posttreatment, the patient demonstrated significant improvement in appetite and taste. Atrophic glossitis, upper limb pigmentation, and frequency of diarrhea also notably decreased. reexamination through endoscopy after 3 months of treatment revealed a substantial decrease in the number and size of gastrointestinal polyps.

**Lessons:**

In this case, from the lower esophageal sphincter to the rectum, there is an increasing trend of eosinophil and mast cell infiltration. These lesions can cause a positive IgG result. Pathological analysis indicates that the extent and severity of lesions in the middle and lower gastrointestinal tract are more substantial than in the upper tract. During treatment, endoscopic observations reveal that lesions in the middle and lower tract tend to resolve faster than those in the upper tract. Hormone therapy has demonstrated significant efficacy in treating this disease. Early treatment and regular follow-up for this disease can reduce the risk of cancerous changes and related complications.

## 1. Introduction

The Cronkhite-Canada syndrome (CCS) primarily manifests as gastrointestinal polyposis, accompanied by diarrhea, hypoproteinemia, and ectodermal changes, including hyperpigmentation of the skin, hair loss, and nail atrophy.^[1]^ CCS is generally considered a rare nonheritable disease.^[2]^ The syndrome typically emerges in older individuals (average age 59 years), with over 80% of patients diagnosed after 50 years.^[3]^ Since the first description of the disease in 1955, approximately 500 cases of CCS have been reported globally, approximately 70% of which are from Japan. Global research the disease is relatively limited, and there is no consensus on treatment approaches.

The diagnosis of CCS primarily depends on typical clinical manifestations, endoscopic examinations, imaging, and pathological results. In this case, the patient had previously undergone partial small intestinal resection due to obstruction. To further substantiate the diagnosis and evaluate the patient’s condition, we conducted comprehensive endoscopic examinations of the stomach, small intestine, and colon. Endoscopic examinations helped observe changes in the mucosa, ulcers, and degree of inflammation.

Furthermore, we obtained tissue samples pertinent to gastrointestinal pathology and used pathological examinations to confirm the CCS diagnosis. Pathological results can show inflammatory changes in the intestinal mucosa, such as lymphocyte infiltration and the formation of cystic fluid pools.

In addition, for posttreatment and evaluation of its effectiveness, we conducted a literature review on CCS. This provides insights into the latest research developments, treatment methods, and prognosis of this disease, thereby guiding clinical practice.

## 2. Case report

### 2.1. Case presentations

A 72-year-old female patient was admitted to the hospital in August 2023 because of chronic diarrhea, reduced appetite, taste alteration, weight loss, and skin changes, including malnutrition of the nails and hyperpigmentation. The patient’s history revealed that she had undergone small intestine resection due to intestinal obstruction 4 years prior. She had been suffering from diarrhea for the past 2 months, with daily bowel movements ranging from to 3 to 4 times, mushy stools accompanied by weight loss, and reduced appetite. Personal history had no history of smoking or drinking, gastrointestinal polyps or surgeries in her family, or endocrine diseases.

### 2.2. Clinical data

All procedures performed in studies involving human participants were in accordance with the ethical standards of the institutional and/or national research committee and with the 1964 Helsinki Declaration and its later amendments or comparable ethical standards. This study was reviewed and approved by the Ethics Committee of North Sichuan Medical College. Informed consent was obtained from the patient for publication of this case report details. After a comprehensive evaluation of the patient’s clinical presentation, histopathology, and endoscopic findings, and by excluding other gastrointestinal diseases, the diagnosis was confirmed as CCS. The purpose of this study is to enhance the understanding and awareness of this disease through a detailed exploration of diagnostic approaches and treatment methods, ultimately aiming to improve patient outcomes.

### 2.3. Physical examination

**Figure 1.**
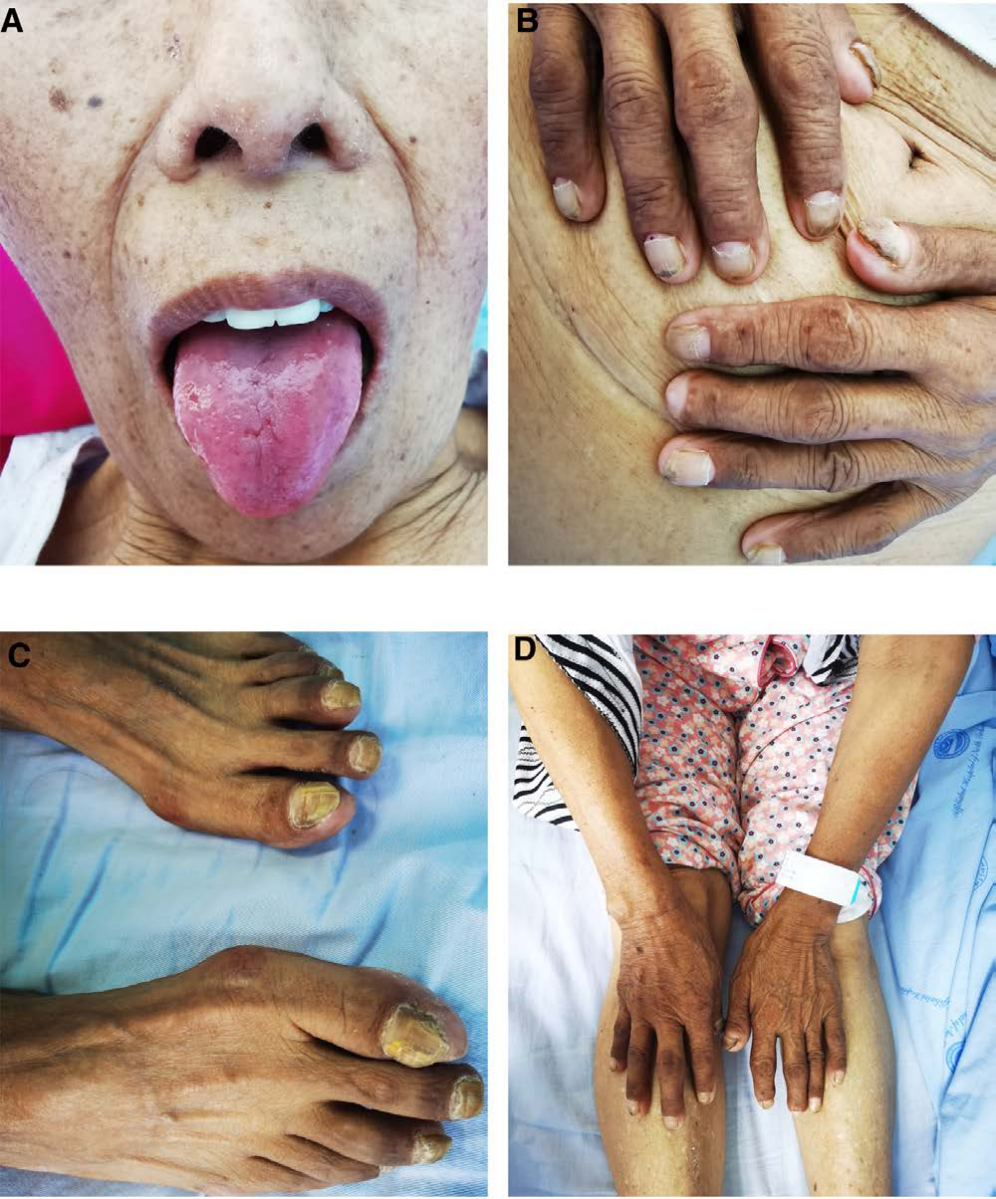
(A) Smooth tongue with atrophic papillae in patient A. (B and C) Nail atrophy in fingers (toes) with distal erosion-like detachment of the nail plate. (D) hyperpigmentation of the skin in the upper limbs.

Physical examination revealed a slender physique, with a height of 152 cm and weight of 43 kg. She displayed skin hyperpigmentation on both upper limbs, malnutrition of the fingernails and toenails on both limbs, fingernail peeling from the proximal to distal end, sparse and shed body hair, glossitis, and atrophy of the papillae (Fig. 1).

### 2.4. Laboratory test

Laboratory examination: EBV(−), CMV(−), HSV(−), TB-SPOT (−), HBsAg (+), HBeAb (+), HBcAb (+), hepatitis B DNA 6*10^3 copies/mL, fecal occult blood (+), cANCA (−), pANCA (−), GBM (−), PR3 (−), MPO (−), ANA (−), normal thyroid hormone levels; IgG 8.29 g/L (7.2–15.6 g/L), IgA 1510 mg/L (800–4530), IgM 593 mg/L (460–3040), IgE 6.13 IU/mL (0–165), IgG4 0.086 g/L (0.030–2.010), PTH 88.3 pg/mL (18.5–88), TSH 1.91 µIU/mL (0.5500–4.7800), HbA1c 5.8% (4.00–6.00), fecal occult blood (+), normal blood lipid and coagulation profiles, prealbumin 147.6 mg/L, total protein 60.0 g/L, albumin 33.7 g/L, hemoglobin 142 g/L, CEA 3.0 µg/L (0.00–5.00).

### 2.5. Endoscopic manifestations

**Figure 2.**
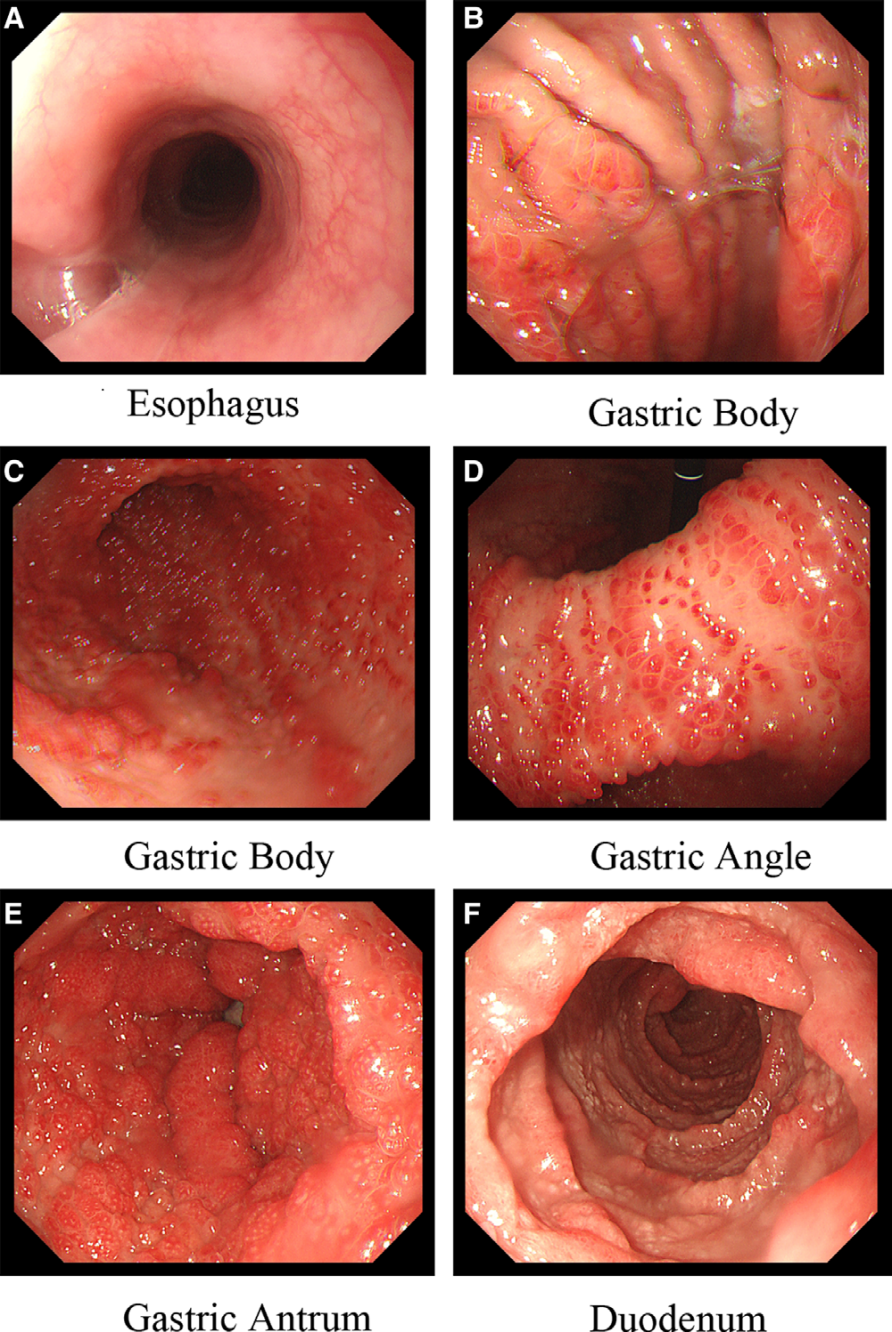
(A) The esophageal mucosa appears smooth with clear vascular pattern. (B–E) The transition from the esophagus to the stomach reveals gradually appearing diffuse nodular, granular, and polypoid elevations on the gastric mucosa. The surface appears smooth, with a reddish color and some exhibiting a strawberry-like appearance. The mucosa shows evident congestion and edema. (G) In the duodenum, scattered polypoid elevations are observed, along with mucosal congestion, edema, and white plaques.

Endoscopy revealed smooth esophageal mucosa with clear vascular patterns. Throughout the stomach mucosa, there were diffuse nodular, granular, and polypoid elevations with a smooth surface, reddish color, partial strawberry-like appearance, and the surface mucosa was significantly congested and edematous. The duodenal mucosa was congested and edematous, with white speck-like changes, diffuse polypoid elevations, and a slightly reddish mucosal surface (Fig. 2).

**Figure 3.**
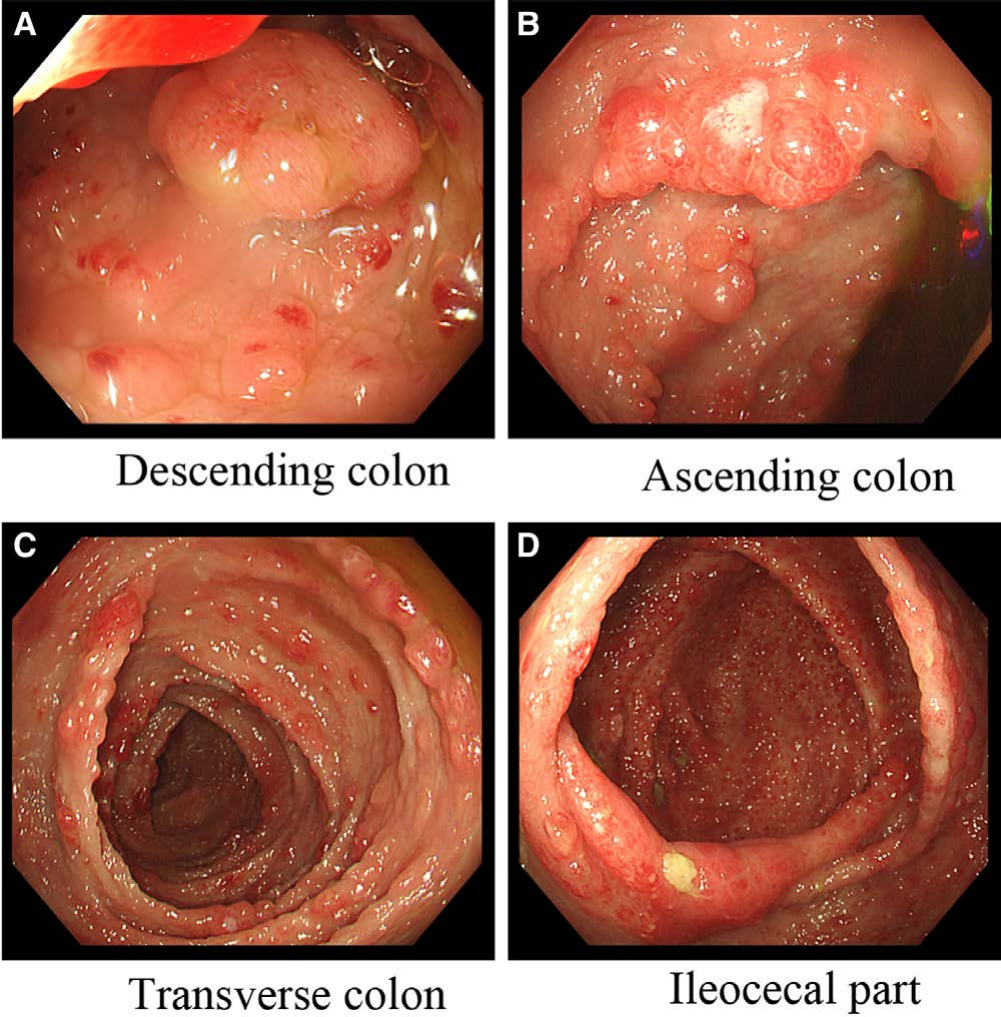
(A–D) The distal ileum mucosa shows mucosal congestion with scattered multiple polypoid elevations characterized by redness and soft biopsy texture. The mucosa of the entire colon and rectum exhibits diffuse polypoid and granular elevations with surface redness and scattered whitening changes. Some have a strawberry-like appearance characterized by oval or finger-shaped shapes, wide base, and inconspicuous stalk. They are distributed diffusely and carpet-like. The mucosal surface appears congested and swollen, and biopsy samples from different locations have a soft consistency. They vary in size, with the smaller ones measuring 4 mm and the larger ones measuring 20 mm.

Colonoscopy revealed diffuse polypoid, granular elevations, a reddish surface on the entire colonic and rectal mucosa, scattered surface whitening changes, polyps of varying sizes, and some with a strawberry-like appearance, ranging in size from 4 mm to 20 mm. The polyps were oval, digitate, with a wide base, lacked a pedicle, had unclear boundaries with the mucosa, and the head end was congested and edematous (Fig. 3).

**Figure 4.**
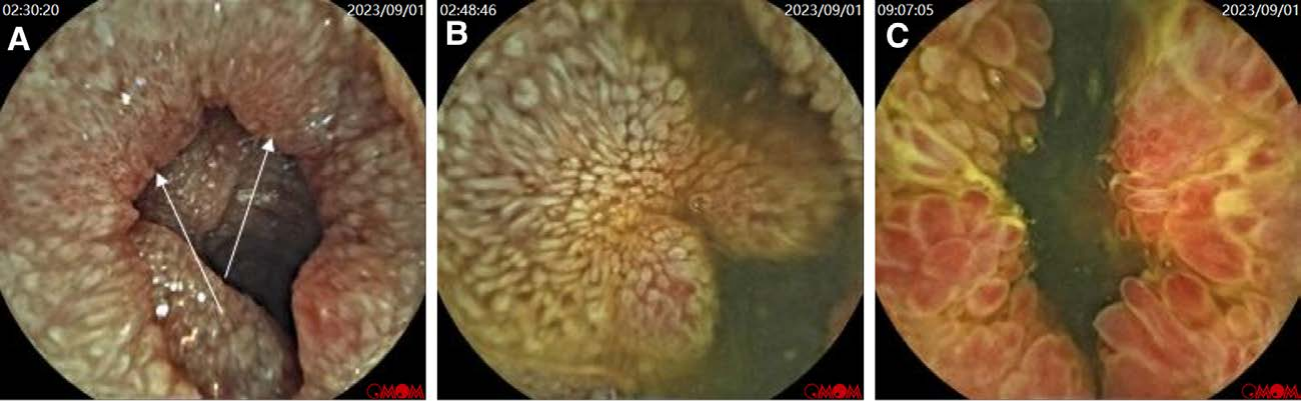
(A–C) The capsule was observed to cause extensive swelling of the small intestine villi in the anterior section, resulting in a change in color to white. In the lower section of the small intestine, the lumen became narrower, and the mucosa exhibited widespread swelling, congestion, and redness, with the villi surface appearing significantly raised, resembling a grape-like appearance with both redness and white color change. Near the ileocecal part, the small intestine lumen exhibited diffuse small polyp-like elevations.

Capsule endoscopy revealed widespread villous swelling in the upper part of the small intestine inside the capsule, narrowing in the lower end of the small intestine, widespread mucosal swelling, congestion, reddening, obvious protrusions on the villous surface resembling grape-like changes, and the surface was visibly congested and red. Close to the ileocecal part, the lumen of the small intestine showed diffuse small polypoid elevations (Fig. 4).

### 2.6. Histopathological examination

**Figure 5.**
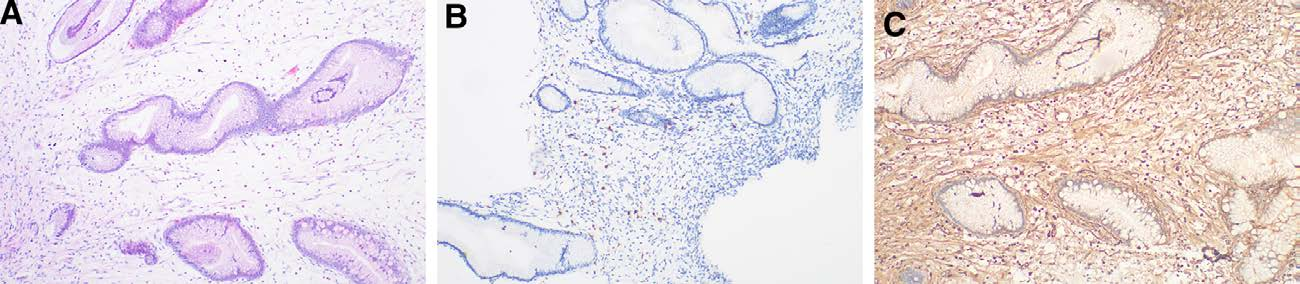
Gastric tissue: (A) (HE ×100) In gastric histopathological samples, mild chronic inflammation, interstitial edema, glandular dilation, and eosinophil infiltration (>15/HPF). (B) (IHC ×100) In the histological examination of gastric tissues, CD117 staining revealed infiltration of mast cell (18/HPF). Additionally, enlarged glandular structures and accumulation of fluid were observed. (C) (IHC ×200) IgG(+) (HE = hematoxylin and Eosin staining; HPF = high-power field; IHC = Immunohistochemistry staining.

Stomach: mild chronic inflammation in the mucosa, interstitial edema, glandular dilatation, intrinsic layer edema, and scattered eosinophil infiltration. The gastric body, antrum, and angle had eosinophil counts of 13, 15, and 16, respectively, per HPF. Mast cell counts at the gastric angle were 18 per HPF (Fig. 5).

**Figure 6.**
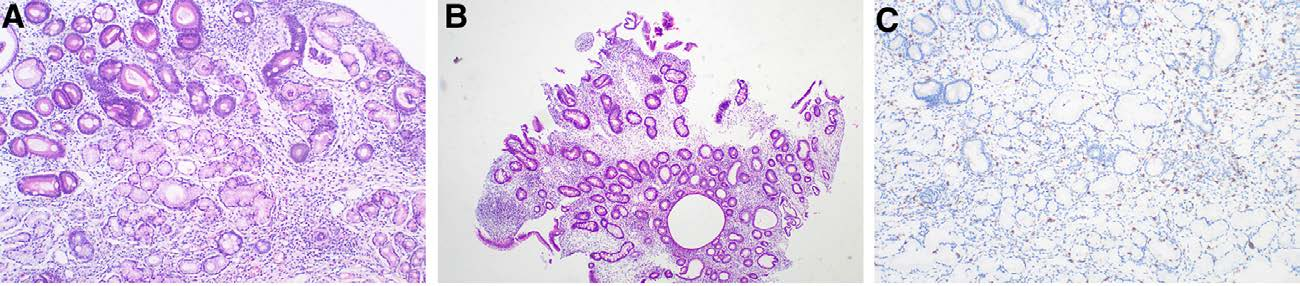
Duodenum (A) (HE ×100), (B) (HE ×40). The mucosa exhibits moderate chronic inflammation, interstitial edema, glandular enlargement, and infiltration of eosinophils (>22个/HPF). (C) (IHC ×100) CD117 staining reveals the presence of mast cell infiltration, with a quantity exceeding 18 per high-power field. Furthermore, the enlargement of glands and accumulation of fluid in cysts are also observable.

Duodenum: Moderate chronic inflammation in the mucosa, interstitial edema, dilatation of individual glands, positive IgG, negative IgG4, slight glandular atrophy, and intrinsic layer edema. The duodenal bulb and descending duodenum had eosinophil counts of 22 and 32 per HPF respectively. Mast cell counts were 34 per HPF and 26 per HPF (Fig. 6).

**Figure 7.**
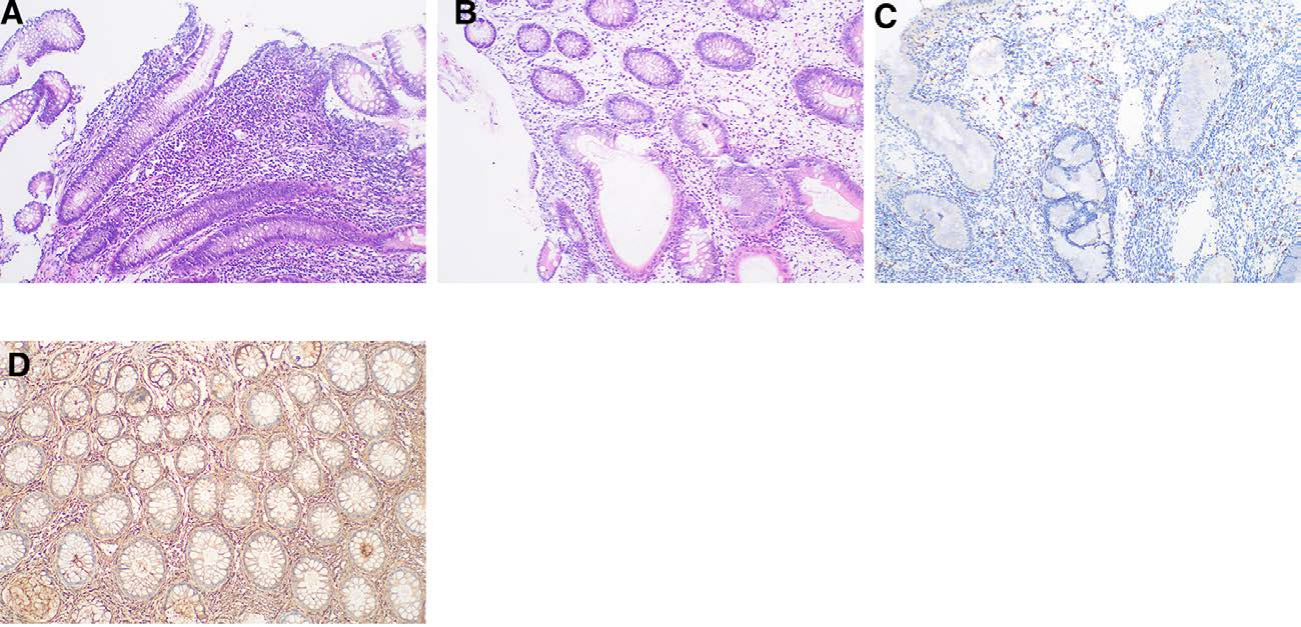
(A) (HE ×100) colonic tissue, scattered infiltrates of eosinophils (>26/HPF) and lymphocytes were observed. (B) (HE ×100). It is evident that the glandular sacs have expanded and formed cystic fluid pools, and it is also visible that the crypts have atrophied and decreased in size. (C) (IHC ×100) CD117 positive, showing infiltration of mast cell (27–29 cells/HPF). (D) (IHE ×100) IgG(+).

Colon and rectum: chronic inflammation with edema in the mucosa, glandular dilatation, and lymphocyte and eosinophil infiltration in the interstitium. Positive IgG and negative IgG4 levels The ileocecal portion, terminal ileum, ascending colon, transverse colon, descending colon, sigmoid colon, and rectum had eosinophil counts of 26, 29, 42, 28, 34, and 26 per HPF, respectively. The ileocecal portion, transverse colon, and sigmoid colon showedmast cell infiltration with counts of 27, 25, and 29 per HPF, respectively (Fig. 7).

### 2.7. Diagnosis and treatment

The case showed that from the lower region of the cardiac orifice to the rectum, eosinophilic and mast cell infiltration showed an increasing trend from small to large, with the lower digestive tract lesions being more extensive than those in the upper and middle digestive tracts.

The patient was diagnosed with CCS in conjunction with pathological, laboratory, and endoscopic examinations, the patient was diagnosed with CCS. Tuberculosis, Epstein-Barr virus, cytomegalovirus, and herpes simplex virus infections were ruled out. In this case, H2RA Ranitidine was used to inhibit gastric acid and antihistamine treatment. Simultaneously, we provided trace element supplements with zinc sulfate and nutritional support, including amino acids, to the patient. The patient’s reduced appetite and taste significantly improved within 4 to 5 days of treatment. Owing to the replication of the patient’s HBV DNA, we adjusted the treatment plan according to the patient’s clinical symptoms, gastroenterological examination, and pathological results. The adjusted regimen consisted of prednisone 40 mg/day, esomeprazole 40 mg/day, loratadine 5 mg/day, mesalazine 1000 mg/day, and Entecavir 0.5 mg qd. After a week of medication, the patient experienced improvement in diarrhea and upper limb pigmentation.

### 2.8. Follow-up

**Figure 8.**
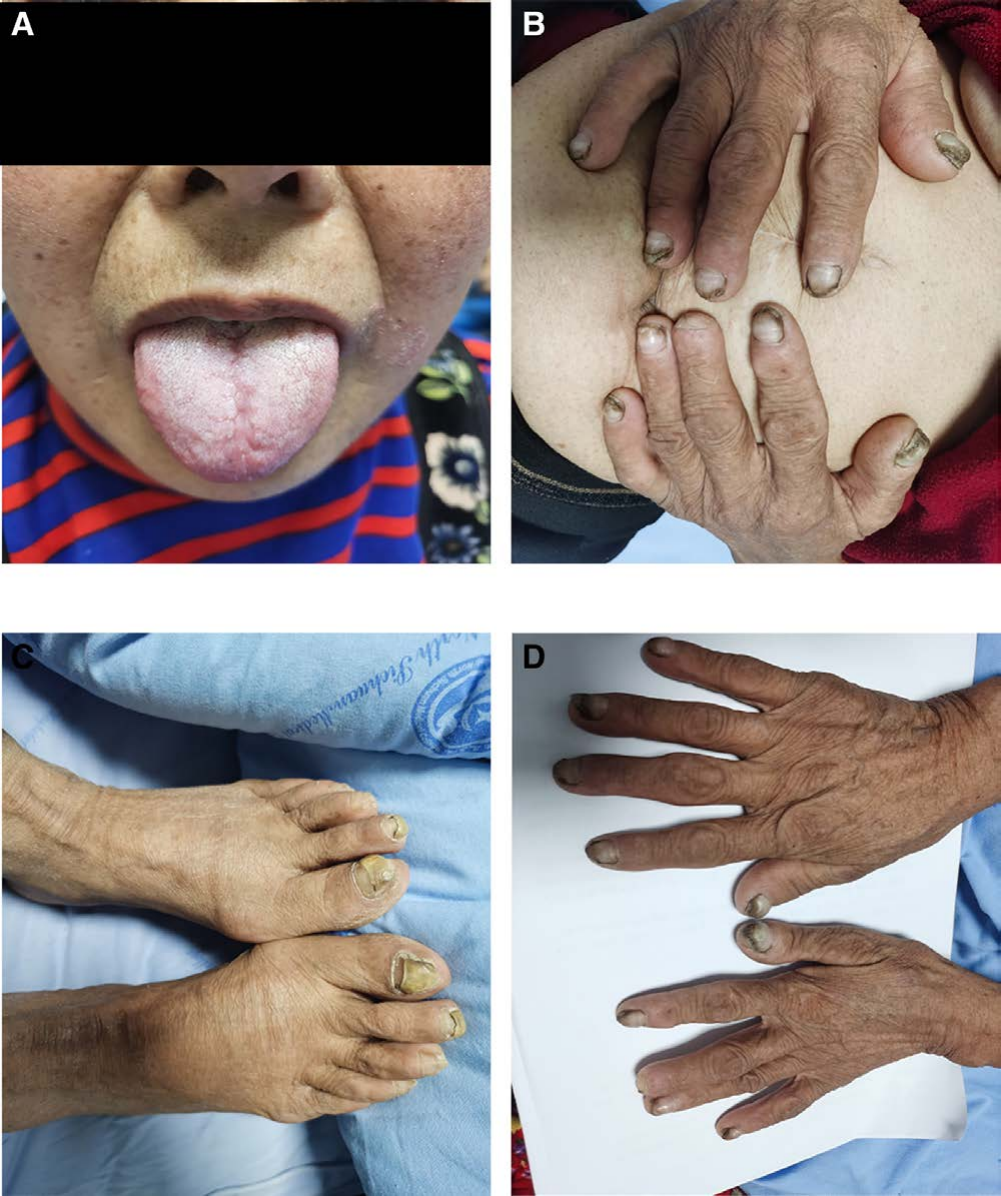
After treatment: (A) compared to Figure A, the patient shows significant improvement in glossitis and atrophy of lingual papillae. (B and C) The patient has newly formed nails. (D) The hyperpigmentation in the patient’s upper limbs has noticeably lightened.

**Figure 9.**
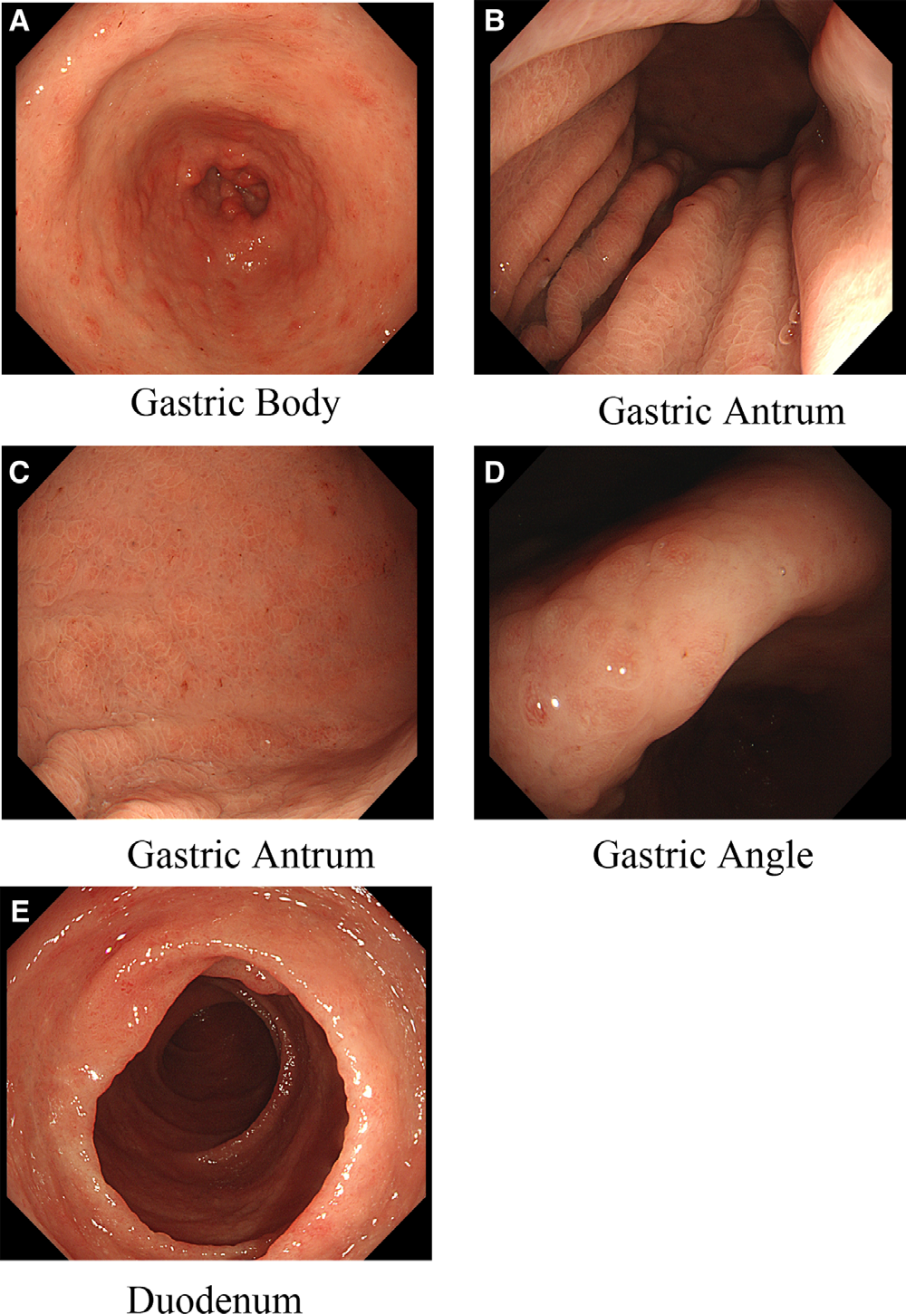
After treatment, there is a significant decrease in the number and size of polypoid lesions in the stomach and duodenum of patients.

**Figure 10.**
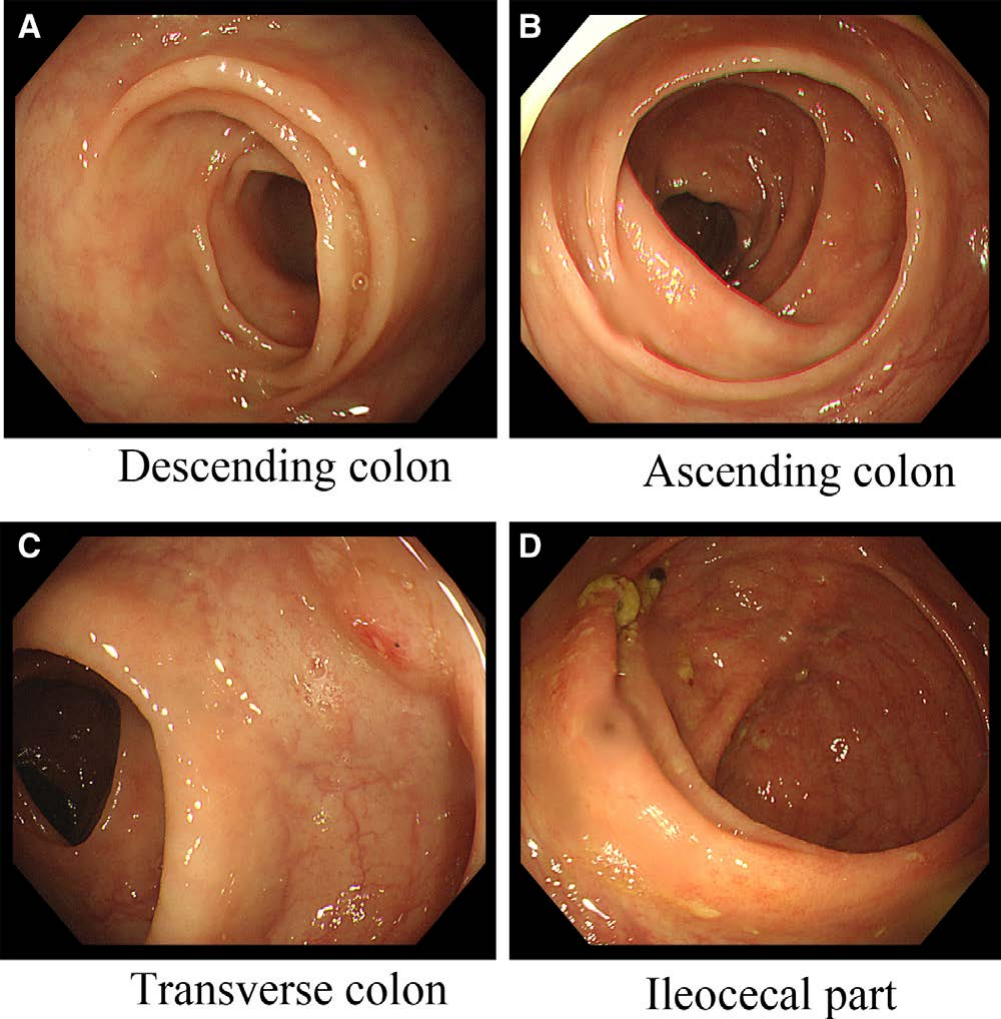
After treatment, there is a significant decrease in the number and size of polypoid lesions in Colon and rectum.

**Figure 11.**
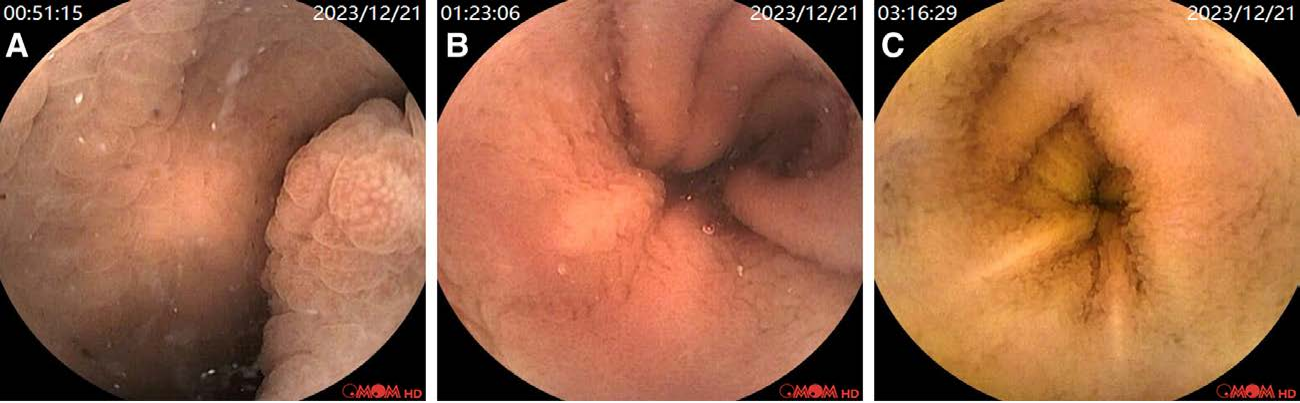
After treatment, the number and size of polypoid lesions in the distal ileum have significantly reduced, and the villous structures in the small intestine are clearly visible.

After treatment, the patient’s appetite, atrophic tongue, and glossitis significantly improved, and her upper limb pigmentation was significantly alleviated (Fig. 8). Three months later, further endoscopic and capsule endoscopic examinations indicated significantly fewer and smaller polyps (Figs. 9–11), and the steroid dosage began to decrease, reducing by 5 mg each week, and then maintained at 5 mg for ongoing treatment.

## 3. Discussion

### 3.1. Clinical manifestations

The most common gastrointestinal symptoms associated with CCS include diarrhea, weight loss, abdominal pain, anorexia, nausea, and hypoproteinemia. In addition, there is notable peripheral edema, glossitis, dry mouth, anemia, and protein-losing enteropathy. Furthermore, electrolyte imbalances, such as calcium, potassium, and magnesium, as well as abnormal sensations, seizures, and limb spasms (associated with electrolyte imbalances and micronutrient deficiencies) may occur.^[4,5]^ While CCS primarily affects the gastrointestinal mucosa, it also presents with ectodermal manifestations, such as alopecia (scalp, eyebrows, pubic hair, armpits, and limbs), nail abnormalities (thinning, splitting, separation from the nail bed starting from the proximal end), and skin hyperpigmentation. The latter can be diffuse or localized, often involving the limbs (especially the hands, and sometimes the palms and soles), face, abdomen, and buccal mucosa, characterized by light to deep brown macules, and in some cases, even vitiligo-like skin changes.^[6,7]^ Other symptoms include hyposmia, cataracts, thrombosis, coagulation disorders, heart failure, peripheral neuropathy, vestibular dysfunction, recurrent acute pancreatitis, and psychiatric disorders.^[4,7]^ Additionally, protein-losing enteropathy, electrolyte imbalances, and non-adenomatous cystic polyps are associated with increased risks of CCS.^[7]^

According to symptoms, Japanese scholars have classified CCS into 5 types: Type I, initial symptoms of diarrhea; Type II, with a decrease in taste as the initial symptom; Type III, with thirst or abnormal oral sensations as the initial symptoms; Type IV, with abdominal discomfort other than diarrhea as the initial symptom; and Type V, with hair loss as the initial symptom. The most common initial symptom of CCS is decreased taste, which may be associated with zinc deficiency. Some patients with taste loss have reported improvement after zinc supplementation, and gastrointestinal symptoms are often related to changes in the gastrointestinal mucosa.^[8–11]^

In addition, some patients have thyroid dysfunction and elevated CEA levels (without evidence of tumors).^[4]^ They may also have systemic lupus erythematosus, rheumatoid arthritis, systemic sclerosis, or LGG4-related autoimmune diseases.^[12,2,7]^

### 3.2. Pathogenesis

Related studies have found that adenomatous foci carrying tumors can be detected in the colon of CCS patients, and all early colon cancers are found to be close to adenomas. Although these histopathological findings are consistent with the adenoma-carcinoma sequence driving malignant tumors in CCS, abnormalities in the lamina propria, such as abnormal crypts or chronic inflammation, may increase the malignant potential through inflammation-induced mutations.^[13–15]^

In all reported cases of CCS, no clinical manifestations or gastrointestinal lesions have been found in first-degree relatives within 3 generations, suggesting a low possibility of a genetic correlation in the pathogenesis of CCS. In a study by Sweetser,^[16]^ elevated levels of IgG4 were found in some CCS patients with polypoid lesions, indicating the infiltration of IgG4 plasma cells in CCS polyps, supporting an autoimmune process. Additionally, this disease has shown effective treatment with steroids and immunosuppressants, further supporting the involvement of autoimmune mechanisms.^[7,17,18]^ In related reports on CCS, immunohistochemical staining of CCS gastrointestinal tissue showed complete positivity for tumor necrosis factor (TNF) in macrophages and lymphocytes.^[19]^ In this particular case of CCS, the patient’s immunoglobulin levels and ANA levels were not elevated, but the gastrointestinal tissue showed positive staining for IgG and infiltration of eosinophils, along with infiltration of mast cells. Currently, the underlying pathogenesis of CCS is considered autoimmune-related.

In a retrospective study by Freeman K,^[5]^ gastrointestinal pathological findings in patients with CCS suggested morphological changes indicative of a low transformative state, namely true atrophy such as glandular disarray, cystic dilatation, loss of specialized cells, and increased connective tissue. He suggested that the aforementioned changes may be due to failure in epithelial or tissue synthesis or their suppression of release. Watanabe-Okada E also suggests that the occurrence of CCS is similar to an alteration in crypt cell differentiation and changes in intestinal mucin production.^[20]^

Allergic reactions are caused by the activation of numerous mast cells. The mechanism of mast cell activation may involve IgE and non-IgE-mediated triggers, clonal mast cell diseases, or may be idiopathic, and can be altered by various factors including, but not limited to, hormonal status, stress, genetic factors, mast cell burden, and simultaneous exposure to more than 1 factor.^[21]^ The sudden release of biologically active mediators into the bloodstream is responsible for the allergic responses.

There is evidence of mast cell infiltration in the colonic mucosa of patients,^[22]^ although mast cells are typically present throughout the gastrointestinal tract. In the gastrointestinal tract, activation of mast cells stimulates the release of mediators, such as histamine and prostaglandins, which regulate intestinal motility and nociceptive sensation. These products regulate epithelial and vascular permeability by promoting the secretion of proteases and pro-inflammatory cytokines (such as TNF-alpha). Furthermore, mast cells contain proteins with antimicrobial and antiparasitic properties, contributing to the maintenance of mucosal barrier function, while mast cells in the muscularis propria primarily interact with nerve plexuses, releasing histamine and proteases to stimulate muscle contractions.^[23]^

Research on mast cells in CCS is limited, and there are currently no detailed studies on the follow-up of CCS diagnosis and treatment at different stages. Currently, there are no established reference ranges for mast cell counts, nor are there acceptable diagnostic thresholds for nonneoplastic mast cell disorders based on mast cell counts,^[23]^ providing a good research direction for future studies and pathological diagnostic criteria.

In addition, genetic susceptibility factors, gene mutations, Helicobacter pylori infection, psychological stress, fatigue, stress reactions, and deficiencies in trace elements may trigger CCS.

### 3.3. Diagnosis

Endoscopically, polyps in CCS are typically diffuse and involve the entire gastrointestinal tract except for the esophagus. Occasionally, the stomach, small intestine (especially the jejunum and proximal ileum), or colon may be selectively affected. CCS gastrointestinal polyps typically have a broad-based, sessile appearance with a cystic or semitransparent characteristic. They vary in size from a few millimeters to 1.5 centimeters and are disorganized, resembling a grape-like mole.^[6,7]^However, a recent study by Tang^[24]^ mentioned esophageal involvement in CCS lesions. In a report by Erno,^[4]^ polyps were found in the gastrointestinal tract of all 32 CCS patients undergoing endoscopy, excluding the esophagus. However, in a study by Watanabe,^[14]^ 26 cases (12.3%) of CCS had esophageal involvement, and most esophageal biopsies showed nonspecific inflammation, with some showing squamous papillomas. In our cases, CCS patients had diffuse polyps in the gastrointestinal tract, including the stomach, duodenum, small intestine, colon, and rectum, excluding the esophagus.

Histologically, CCS polyps exhibit a hamartomatous appearance with sessile polyps without a stalk. The lamina propria shows edema and expansion, with infiltration of a few single-nucleated inflammatory cells, as well as distorted, expanded, and proliferating cystic glands (some of which are cystically dilated, filled with proteinaceous fluid or concentrated mucus) or crypts. The mucosa often contains congested blood vessels, surface erosion, and infiltration of eosinophils,^[4,7,16,25,26]^ relevant reports, scattered smooth muscle fibers,^[26]^ smooth muscle cells, significant mast cell infiltrates, and infiltration of IgG4 plasma cells (8) have been observed in the pathological tissues.^[7]^

### 3.4. Treatment

Due to the rarity of CCS and the lack of understanding of its etiology, there is currently no consensus on the treatment approach for CCS. Many drugs have been used in the treatment of CCS, often in combination, including nutritional supplements, antibiotics, and corticosteroids, which are the most commonly used. However, histamine receptor antagonists and sodium cromoglycate have shown promising results.^[22]^ The treatment approach for CCS can be summarized as follows:

1) Nutritional support: CCS significantly affects the gastrointestinal tract, leading to polypoid proliferation and digestive disorders, such as malabsorption, gastrointestinal bleeding, and protein loss. Therefore, nutritional support is essential, particularly for patients with severe malabsorption and weight loss. Supplementation with electrolytes, trace elements, amino acids, vitamin B12, folic acid, etc, is recommended. Albumin supplementation can facilitate intestinal mucosal repair.
2) Hormonal therapy Corticosteroid therapy is the preferred approach for drug-induced CCS. A study conducted by Watanabe C^[14]^ demonstrated that corticosteroids alleviate abdominal symptoms within a few months, but polyp regression usually takes more than 6 months. Diarrhea symptoms improved within 51 days, taste reversal required 84 days, ectoderm changes required 97 ± 11.2 days, and a reduction in polyp size and number occurred at a slower pace. Gastric polyps require an average of 248 days, whereas colon polyps require an average of 238 days.^[14]^ Daily oral administration of prednisone at a dose of 30 to 49 mg is recommended for CCS treatment. However, rapid reduction of steroid dosage may result in early relapse; therefore, gradually tapering the prednisolone dose after endoscopic confirmation of polyp regression is advised.^[14]^ CCS typically prefer hormone therapy for treatment. However, according to a retrospective study of 9 CCS cases conducted by LI S, it was found that some patients may experience a relapse after discontinuing hormonal treatment, and some may even show no response to hormones.^[27]^
3) Immunosuppressants, such as a zathioprine and cyclosporine A, are commonly used immunosuppressants. In a study conducted by Onozato Y,^[28]^ a CCS patient experienced relapse during steroid tapering, and reinitiation of steroid treatment led to membranous nephropathy. The addition of cyclosporine A significantly improved proteinuria and residual gastrointestinal polyps. Therefore, if steroids are not effective, relapse occurs after steroid tapering, or long-term steroid maintenance is needed, combination therapy with these drugs should be considered.
4) Antimicrobial drugs: According to a study by Daniel E al,^[4]^ 2 patients diagnosed with CCS based on diarrhea symptoms exhibited improvement when treated with antibiotics. However, antibiotic treatment is not commonly employed for CCS, except when patients have concurrent intestinal infections, to promote recovery and reduce the risk of complications such as intestinal mucosal damage and infection.
5) Surgery: Although CCS is not a tumor, long-term inflammatory reactions and stimuli increase the risk of gastrointestinal tissue malignancy. Therefore, regular gastrointestinal endoscopy is necessary for the early detection and removal of high-risk gastrointestinal polyps. Surgery is indicated in patients with intractable peptic ulcers, gastrointestinal bleeding, intestinal obstruction, intestinal intussusception, and gastrointestinal malignancies.
6) Anti-allergy treatment: Mast cell infiltration was observed in the colonic mucosa of a patient with CCS. Mast cell proliferation or migration to gastrointestinal tissue can result in increased circulation of cytokines and other substances, potentially contributing to the development of CCS, as suggested by Ward EM.^[22]^ Patients with CCS should undergo evaluation for potential mast cell dysfunction, as proposed by Ward. If mast cell dysfunction is detected, sodium cromoglycate (200 mg, q.i.d.) can be administered as a treatment option. Mast cell dysfunction can be detected through mucosal biopsy specimens, as well as elevated levels of histamine or its metabolites in the serum or urine.^[22]^ Despite the scarcity of research on mast cells in CCS, the precise relationship between mast cells and CCS remains unclear. However, it is possible to speculate on other gastrointestinal diseases associated with mast cells.
7) Traditional Chinese medicine treatment: In a study conducted by Hu H,^[29]^ a CCS patient declined steroid treatment and switched to traditional Chinese medicine and nutritional support. The patient experienced improvements in diet, mental condition, and relief from diarrhea after 1 week. Subsequent examinations revealed improvements in anemia and nutritional indicators, with the immune indicators approaching normal levels. The patient’s discomfort resolved during subsequent treatment. Traditional Chinese medicine contains steroid-like substances and certain ingredients that can regulate the immune system, provide essential trace elements, and enhance gastrointestinal motility.^[29]^
8) TNF: Reports suggest that a CCS patient underwent treatment with corticosteroids, anti-fibrinolytic agents, and mercaptopurine, but the treatment proved ineffective, resulting in persistent symptoms and intestinal intussusception.^[30]^ According to a report, CCS tissue exhibited elevated levels of TNF-alpha (TNF-α).^[19]^ Consequently, the patient initiated induction therapy against the TNF by receiving 200 mg of infliximab every 2 weeks. Colonoscopy conducted 20 months after induction therapy revealed complete remission and disappearance of the polyps. Subsequently, the patient has been receiving 200 mg of infliximab every 8 weeks and has remained symptom-free for 3 years since the initial administration. Furthermore, Taylor SA^[12]^ reported a notable improvement in clinical symptoms among CCS patients who were unable to tolerate hormones and immunosuppressants when infliximab was administered.
9) Salicylic acid preparations: In a report by Takakura M,^[31]^ a female patient was diagnosed with CCS based on various clinical manifestations, including diffuse hair loss, nail deformities, reduced taste sensation, skin pigmentation, abdominal discomfort accompanied by diarrhea, and weight loss. Initially, the patient underwent high-nutrient corticosteroid therapy. This combination therapy alleviated the associated clinical symptoms, abdominal symptoms, and diarrhea resurfaced upon implementing an elemental diet and tapering steroidal treatment. Endoscopy and histological examination revealed persistent inflammation, especially in the colon. Consequently, a combination therapy of mesalazine and steroids was initiated, leading to substantial alleviation of abdominal pain, a decrease in the frequency of diarrhea within a few days, and complete regression of colon polypoid lesions after 3 months.
10) Anti-Helicobacter pylori therapy Anti-Helicobacter pylori treatment resulted in significant improvement in clinical symptoms, ectodermal manifestations, and endoscopic gastrointestinal polyps in 2 cases with CCS.^[32,33]^

### 3.5. Prognosis

According to the current research, the prognosis of CCS is poor, with a 5-year mortality rate of up to 55%. Complications such as gastrointestinal bleeding (mainly due to erosion and ulceration of polyps), intussusception, intestinal obstruction, and rectal mucosal prolapse are common causes of death.^[4]^ Malnutrition, hypoalbuminemia, recurrent infections, sepsis, heart failure, and gastrointestinal bleeding are commonly observed in CCS-related mortality cases.^[5,27]^

## 4. Conclusions

Although CCS is not a tumor lesion, it is associated with a certain risk of carcinogenesis. According to Shuang Liu research,^[2]^ a patient was diagnosed with rectal cancer 5 years after CCS diagnosis, and the surrounding mucosa appeared normal. Patients with CCS usually respond well to corticosteroids, with an increased 5-year survival rate. The size of gastric polyps is associated with poorer survival rates and non-recurrence survival rates, while being over 60 years of age is another predictive factor for worsened non-recurrence survival rates. After treatment, diffuse gastrointestinal polyps may partially or completely disappear, and the response to colonic lesions is better than that to gastric lesions.^[2]^

Since CCS mainly affects the digestive tract outside the esophagus, the stomach, duodenum, small intestine, and colon may present with symptoms such as polyp erosion and bleeding under inflammatory manifestations, and even malignant polyps or gastrointestinal malignancies. A study followed up 383 patients with CCS, of whom 91 had concurrent tumors.^[14]^ Therefore, CCS requires long-term endoscopic follow-up. Steroid use is beneficial for controlling inflammatory reactions. NBI, magnifying endoscopy, and dye-based contrast agent-enhanced techniques can help to identify and remove precancerous or malignant lesions. In a study by Watanabe C,^[14]^ CCS patients showed endoscopic recurrence 1 year earlier than clinical symptomatic recurrence after discontinuing steroid hormones.

Abdominal symptoms are relieved within a few months after treatment with corticosteroids, while polyp regression usually takes more than 6 months. Compared with relapsers or nonresponders, maintaining endoscopic remission with corticosteroids significantly reduces the development of CCS-related cancers. This highlights the importance of sustained endoscopic remission in cancer prevention.^[14]^ Therefore, early diagnosis and effective treatment are crucial to prevent complications, posing a major challenge for clinical physicians. This article provides a general overview of its diagnosis, clinical manifestations, endoscopic and pathological findings, and treatment experience, hoping to be helpful for clinical physicians in their work.

## 5. Limitations

This article meticulously explores a case report dealing with CCS, a rare gastrointestinal condition. The conclusions drawn from this single-case study may not be widely generalizable to all individuals afflicted with CCS, presenting a noteworthy limitation. Additionally, the rarity of CCS poses considerable obstacles to conducting statistical analysis with large and representative samples. Furthermore, the absence of a control group or controlled variables within the study framework makes it difficult to firmly establish causal connections regarding the efficacy of the treatment. The long-term outcomes and safety profile of the proposed treatment regime have also not been sufficiently evaluated. Despite these constraints, the findings of this investigation provide valuable insights for further research and management of CCS. They also establish a foundational basis for future studies aimed at developing more effective treatment modalities, thus contributing to the broader discourse within the field.

## Acknowledgments

We extend our sincere gratitude to JINSHAN Science & Technology for their support in providing the SC-100 small intestine capsule endoscope.

## Author contributions

**Conceptualization:** Wen Ming, LIjun Ren, Tao Huang, Peng Zhu.

**Data curation:** Yue Xiang, Peng Zhu, Min Huang.

**Formal analysis:** Yue Xiang, Jian Gao, GuoDong Yang, Min Huang, Quan Ren.

**Funding acquisition:** Yue Xiang, Liping Tao, GuoDong Yang.

**Investigation:** Liping Tao.

**Methodology:** Jian Gao.

**Writing – original draft:** Nanping Wang.

**Writing – review & editing:** Nanping Wang.
